# Human 3D Airway Tissue Models for Real-Time Microscopy: Visualizing Respiratory Virus Spreading

**DOI:** 10.3390/cells11223634

**Published:** 2022-11-16

**Authors:** Marion Möckel, Nino Baldok, Thorsten Walles, Roland Hartig, Andreas J. Müller, Udo Reichl, Yvonne Genzel, Heike Walles, Cornelia Wiese-Rischke

**Affiliations:** 1 University Clinic for Cardiac and Thoracic Surgery, Otto-von-Guericke-University Magdeburg, D-39120 Magdeburg, Germany; 2 Bioprocess Engineering Group, Max Planck Institute for Dynamics of Complex Technical Systems, D-39106 Magdeburg, Germany; 3 Institute for Molecular and Clinical Immunology, Otto-von-Guericke-University Magdeburg, D-39120 Magdeburg, Germany; 4 Helmholtz Centre for Infection Research, D-38124 Braunschweig, Germany; 5 Core Facility Tissue Engineering, Otto-von-Guericke-University Magdeburg, D-39106 Magdeburg, Germany

**Keywords:** human 3D airway tissue model, respiratory virus, virus spreading

## Abstract

Our knowledge about respiratory virus spreading is mostly based on monolayer cultures that hardly reflect the complex organization of the airway epithelium. Thus, there is a strong demand for biologically relevant models. One possibility to study virus spreading at the cellular level is real-time imaging. In an attempt to visualize virus spreading under somewhat more physiological conditions, Calu-3 cells and human primary fibroblasts were co-cultured submerged or as air-liquid interface (ALI). An influenza A virus (IAV) replicating well in cell culture, and carrying a red fluorescent protein (RFP) reporter gene was used for real-time imaging. Our three-dimensional (3D) models exhibited important characteristics of native airway epithelium including a basement membrane, tight junctions and, in ALI models, strong mucus production. In submerged models, first fluorescence signals appeared between 9 and 12 h post infection (hpi) with a low multiplicity of infection of 0.01. Virus spreading further proceeded in the immediate vicinity of infected cells. In ALI models, RFP was found at 22 hpi and later. Consequently, the progression of infection was delayed, in contrast to the submerged model. With these features, we believe that our 3D airway models can deliver new insights in the spreading of IAV and other respiratory viruses.

## 1. Introduction

Our current understanding of respiratory virus infection and spreading is largely based on two-dimensional (2D) monolayer cell cultures or animal models (e.g., [1,2]). 2D monolayer cultivation has significant limitations in mimicking the physiological complexity of the airway tissue in vivo. Therefore, such models may not accurately reproduce the natural infection process. Animal models, in turn, can face the problem of host specificity of the pathogen. They are more expensive and time-consuming, and unknown pathogenic mechanisms cannot be isolated. Additionally, ethical considerations apply and it is not clear whether the results can be translated directly to humans. Three-dimensional (3D) cell culture models could help to overcome these restrictions. There are several different 3D airway models that were developed in the last decade including human lung explants, stem/progenitor cell derived lung organoids, and 3D models composed of primary airway epithelial cells, or cell lines [3]. It has been recognized that biologically relevant 3D airway tissue models should contain components of the extracellular matrix (ECM) and consider the co-cultivation of epithelial cells with fibroblasts [4] as the major cell type of the subepithelial layer. Both of these have been hardly taken into account until now.

Real-time fluorescence microscopy is a powerful tool to study virus spreading in tissues; however, it is not applicable in the existing models [5,6].

Therefore, we generated a new, complex 3D airway tissue model affording real-time microscopy of virus infection. We utilized Calu-3 cells that possess many characteristics of differentiated, functional bronchial epithelial cells [7]. They have been successfully used for virus infection studies with influenza A virus (IAV) [8,9]. Calu-3 cells can produce mucus and basement membrane proteins, and build tight junctions to generate an epithelial barrier in a 3D environment [10,11,12]. We co-cultivated these cells with human primary fibroblasts (hpF) on a biological collagen IV rich matrix to obtain epithelial polarization and to mimic the connective tissue and the extracellular microenvironment, respectively.

Airlift (ALI) cultivation supports epithelial cell differentiation including the formation of mucus-producing cells. Submerged and ALI cultivation support the generation of a physiological epithelial barrier. These features have been shown to influence the infection of cells with respiratory viruses [13], and therefore both cultivation methods have been applied in this study.

To establish the real-time fluorescence microscopy, we used a A/PR/8/34 IAV, where the NS1 sequence was fused to the red fluorescence protein (RFP) sequence (NS1-RFP IAV, [14]). The A/PR/8/34 IAV is a well-characterized human influenza virus strain which has been used for experimental studies regarding virus-host cell interactions and virus spreading in cell populations (e.g., [15,16]) and to infect Calu-3 cells [17].

With this study, we have provided a basis for investigating respiratory virus spreading in real time via microscopy. To our knowledge, this is the first study monitoring IAV infection spreading in real time on a cellular level in 3D airway tissue models.

## 2. Materials and Methods

### 2.1. Primary Cells and Tissue

Human primary fibroblasts (hpF) derived from skin were isolated and the bronchus was received from the surgical specimen. The patient’s informed consent was obtained before surgery and the studies were approved by the institutional ethics committee on human research of the Julius-Maximillians-University Würzburg (vote 182/10) and the ethics committee of the medical faculty of the Otto-von-Guericke-University Magdeburg (vote 163/17), respectively.

### 2.2. Cell Culture

Calu-3 cells (American Type Culture Collection (ATCC), Manassas, VA, USA), hpF, and MDCK cells (European Collection of Authenticated Cell Cultures (ECACC), UK Health Security Agency, Salisbury, UK) were cultivated in MEM medium containing GlutaMAX (Thermo Fisher Scientific, Waltham, MA, USA) supplemented with 1 mM sodium pyruvate (Thermo Fisher Scientific), and 10% FBS (Bio&Sell, Feucht, Germany), in DMEM medium (high glucose) containing GlutaMAX and sodium pyruvate (Thermo Fisher Scientific) with 10% FBS, and GMEM medium (Thermo Fisher Scientific) with 1% tryptose No. 2 (NEOGEN, Lansing, MI, USA), and 10% FBS, respectively. Media were changed every two to three days. All cells were maintained under standard culture conditions in a humidified incubator containing 5% CO_2_ at 37 °C.

### 2.3. 3D Tissue Engineering

3D airway tissue models were generated using porcine-derived decellularized small intestinal submucosa (SIS) without serosa representing the physiological ECM [18]. Small pieces of this collagenous scaffold clamped between two metal rings (cell crown) were placed into 12 well plates. DMEM with 10% FBS was added basally (1.5 mL) and apically (1 mL). On day one, 1 × 10^5^ hpF per cell crown were plated onto the matrix and cultured with DMEM containing 10% FBS (see before). We used hpF from one donor to keep this component of the 3D model constant. On day two, 4 × 10^5^ Calu-3 cells per crown were added to assemble submerged 3D airway models. These 3D models were cultivated basally and apically in medium with a ratio of 1:1 of DMEM with 10% FBS, and MEM supplemented with 1 mM sodium pyruvate, and 10% FBS. On day three, ALI cultivation was started by transferring the seeded cell crowns into 6 well plates and supplying 3 mL of the 1:1 medium mixture basally only. All 3D models were maintained under standard culture conditions in a humidified incubator containing 5% CO_2_ at 37 °C. The medium was changed every two to three days.

### 2.4. Histology and Immunofluorescence Staining

3D models were embedded with Tissue Tek O.C.T. compound (Sakura Finetek Europe B.V., Alphen aan den Rijn, The Netherlands) at the respective days and cryo-sectioned at 10 µm thickness. To detect mucus, alcian blue staining was performed according to standard protocols. The staining was also used to histologically estimate the thickness of the mucus layer in the 3D models. For immunofluorescence stainings, sections were fixed with 4% PFA in PBS (pH 7.4) for 10 min at room temperature or acetone for 10 min at −20 °C for CD90 antibody staining. After washing with PBS, sections were treated with blocking/permeabilization solution (PBS containing 3% goat serum, and 0.1% Triton X100 except for CD90 antibody staining) for 30 min at room temperature. This was followed by primary antibody incubation. Antibodies directed against E-cadherin (Cell Signaling Technology, Danvers, MA, USA, clone 24E10, 1:150), ZO-1 (GeneTex, Irvine, CA, USA, GTX108613, 1:100), Muc5AC (Thermo Fisher Scientific, clone 45M1, 1:100), or CD90-FITC (Dianova, Hamburg, Germany, clone AS02, 1:50) were diluted in blocking solution and incubated overnight at 4 °C. Subsequently, secondary antibodies (goat anti-rabbit IgG-Cy3, Jackson ImmunoResearch, West Grove, PA, USA, 1:500, and goat anti-mouse IgG1-Alexa Fluor 488, Jackson ImmunoResearch, 1:500) diluted in blocking solution were incubated for 1 h at room temperature, and nuclei were counterstained with DAPI for 10 min at room temperature. Finally, sections were washed in PBS and embedded in Mowiol 4-88 with DABCO (2.5%, both from C. Roth GmbH, Karlsruhe, Germany). Negative controls (omission of primary antibodies) were performed for each antibody to control the non-specific binding of the secondary antibodies.

### 2.5. Determination of Total Cell Numbers of 3D Airway Tissue Models

3D airway models were washed with PBS, and cells were lysed in Tris buffer (10 mM) pH 8, EDTA (1 mM) with 0.2% Triton X100 for 30 min on ice on the respective day. DNA contents were determined using 1× picogreen reagent in TE buffer (10 mM Tris-HCL, 1 mM EDTA). Samples were excited at 480 ± 9 nm, and the fluorescence emission intensity was measured at 520 ± 20 nm using an Infinite M Plex microplate reader (Tecan, Männedorf, Switzerland). Cell numbers were calculated using a linear standard curve of DNA isolated from samples with defined cell numbers. The total cell number allowed for the estimation of the virus amount to be added corresponding to the desired multiplicity of infection (MOI).

### 2.6. Influenza Virus Strains and Infection

The following IAV strains were used: MDCK adapted seed of A/PR/8/34 (H1N1) (RKI) with a titer of 1.23 × 10^8^ TCID_50_/mL and A/PR/8/34-NS1-RFP with a titer of 2.88 × 10^7^ TCID_50_/mL.

Before infection, 2D cell cultures and submerged 3D models (apical and basal side) were washed twice with PBS to remove viral inhibitors. ALI models were washed twice basally only. For infection, 2D cell cultures in 6 well culture plates were cultivated in (1) MEM containing 1 mM sodium pyruvate (basal medium, “-”), (2) basal medium with 10% FBS (“FBS”), and (3) basal medium with 1% porcine trypsin (“T”), and infected at an MOI of 0.01, 0.1, or 1, respectively. For infection of 3D airway tissue models, basal medium with FBS and an MOI of 0.01, 0.14, or 1 was used. The virus stock was diluted in the respective medium according to the cell number and respective MOI, and mixed. This virus solution was equally disseminated on the cells. After 1 h of incubation for virus binding and entry, the medium was removed and fresh cell type-specific culture medium was added for further incubation and virus replication.

### 2.7. Virus Quantification

2D cell culture supernatants of 250 µL were collected at the respective time points and centrifuged at 550× *g* for 5 min. The virus supernatants were stored at −80 °C until further analysis. Total numbers of virus particles were determined by HA assay as described previously [19]. The HA titers were displayed as log10 (HA units/100 µL) and can be converted into virions/mL according to Isken et al. [20]. The titer of infectious virions was measured using a tissue culture infectious dose 50 (TCID_50_) assay [20]. The TCID_50_ values were calculated according to the method described by Spearman and Kärber [21]. Additionally, RT-qPCR was performed for IAV genome segment 5 according to Kupke et al. and Frensing et al. [22,23]. In 3D submerged models, NS1-RFP-positive cells and Hoechst 33342-positive nuclei (total cell number) were counted on the basis of the images taken during live-cell imaging. The percentage of NS1-RFP-positive cells was calculated with the help of the total number of cells per image.

### 2.8. Microscopy, Real-Time Fluorescence Imaging, and Image Processing

For multidimensional imaging, 3D submerged models were stained with Hoechst 33342 and Calcein-AM in FluoroBrite DMEM medium (Thermo Fisher Scientific) with 1 mM sodium pyruvate for 1 h under standard culture conditions at 37 °C and 5% CO_2_. The dyes were removed and the tissue models were washed three times for 5 min with PBS. The 3D models were then transferred inversely into µ-dishes (ibidi GmbH) and covered with FluoroBrite DMEM medium with 1 mM sodium pyruvate (see above). They were analyzed using an inverted Confocal Microscope System Leica SP8 (Leica Microsystem, Wetzlar, Germany) controlled by LAS X software (Leica Microsystem) and equipped with a Plan Apo 63×/1.4 oil objective. To avoid bleed-through of fluorescence emission, sequential unidirectional scanning was performed at 700 Hz using the following settings. Sequence 1: excitation at 488 nm, emission at 492 nm–528 nm, and sequence 2: excitation at 405 nm, emission at 413 nm–449 nm. Sequences were altered between lines. Voxel size was adjusted to 70 nm × 70 nm × 1576 nm (dx, dy, dz). Images of the channels were pseudo-colored; Hoechst 33342 (excitation 405 nm) in blue, Calcein-AM (excitation 488 nm) in green.

Additionally, two-photon laser scanning microscopy was performed using the TriM Scope II from LaVision BioTec GmbH, Bielefeld, Germany. The samples were scanned with laser power adaption and a pixel size of 435 × 435 nm at a scanning frequency of 400 Hz. The emission of Calcein-AM and Hoechst 33342 were recorded at 525/50 nm and 420/50 nm, respectively, using a PMT detector.

For real-time fluorescence imaging of infected 2D cell cultures and 3D airway models, the cells were stained with Hoechst 33342 for 10 min at 37 °C and 5% CO_2_. After removing the Hoechst stain, in 2D cell cultures, the MEM medium including supplements was added. Infected 3D airway models were transferred inversely into µ-dishes and covered with FluoroBrite DMEM medium with 1 mM sodium pyruvate (see above).

In 2D cell cultures four to seven individual positions were monitored and in 3D airway models eight individual positions were monitored.

Immunostainings of cryo-sections and infected 2D cell cultures were examined using the Evos FL Auto 2 imaging system (Thermo Fisher Scientific) equipped with an onstage incubator and an Olympus Luc Plan FL N 40×/0.6 2.7–4.0 WD, and an Evos Plan FL 40×/0.65 LWD objective, respectively. The following settings were employed: for DAPI: excitation at 357/44 nm and emission at 447/60 nm, for Alexa Fluor 488 or FITC-conjugated antibodies: excitation at 482/25 nm and emission at 524/24 nm, for Cy3-conjugated antibodies and RFP: excitation at 542/20 nm and emission at 593/40 nm.

Real-time fluorescence imaging of infected 3D airway models was performed using either the Zeiss Axiovert 200 M or the Zeiss Axio Observer Z1 with AxioCam MR using AxioVision software, both with a heated live-cell incubation chamber adjusted to 37 °C and 5% CO_2_. An LD C-Apochromat 40×/1.1 W Korr UV VIS IR (Carl Zeiss, Oberkochen, Germany) and a Plan-Neofluar 40×/0.75 Ph2 objective (Carl Zeiss) were used to analyze the cells. The Axiovert 200 M microscope was equipped with filters for RFP signal with excitation at 565/30 nm and emission at 620/60 nm, and for the Hoechst 33342 signal with excitation at 365 nm and emission at 445/50 nm. The Axio Observer Z1 microscope contained the filter set 25 HE DAPI/FITC/Texas Red (Carl Zeiss). Phase-contrast light microscopy of living cell cultures was performed using the Evos XL Core (Thermo Fisher Scientific). Images were processed using ImageJ (version 1.52, https://imagej.nih.gov/ij/index.html (accessed on 22 September 2022)) and Fiji (version released 30 May 2017, https://imagej.net/Fiji (accessed on 22 September 2022)). The 3D rendering of stacks was performed using “Icy” software (version 1.9.6.0, http://icy.bioimageanalysis.org/ (accessed on 22 September 2022)).

### 2.9. Determination of Epithelial Layer Thickness

The epithelial layer thickness of 3D ALI models at days 7 and 14 was determined using cryo-sections stained with alcian blue. ImageJ was used to measure the thickness of the epithelium at three positions per image in up to four images per experiment. The average and standard deviation were calculated from four (for day 7) and five (for day 14) independent experiments, respectively.

## 3. Results

### 3.1. Human 3D Airway Tissue Models Consisting of Calu-3 Cells and Fibroblasts Resemble Bronchial Epithelial Tissue

**Figure 1:**
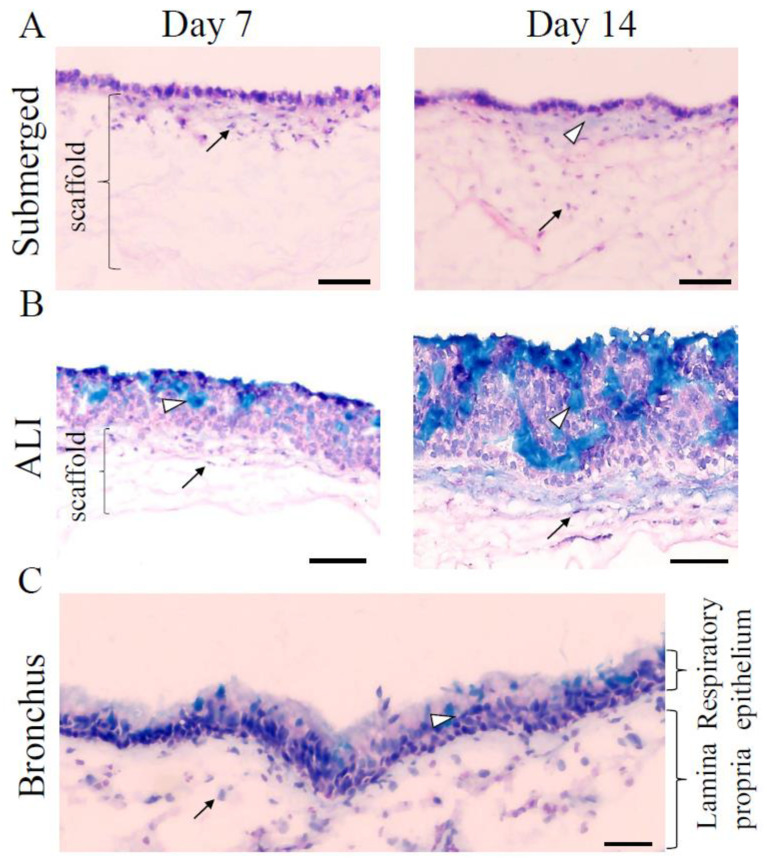
Three-dimensional (3D) tissue engineering produces well-developed 3D airway tissue models within 14 days. (**A**) Alcian blue staining of 3D submerged models cultivated for 7 (**left**) and 14 (**right**) days, respectively. White arrowheads point to a modicum of mucus indicated by the light blue color. (**B**) Alcian blue staining of ALI models cultivated for 7 (**left**) and 14 (**right**) days, respectively, showed the cumulative thickness of the mucus layer (see white arrowheads). Black arrows indicate human primary fibroblasts (hpF) within the collagen scaffold that is marked with a bracket. Three independent experiments were performed. (**C**) Alcian blue staining of the native bronchial epithelium shows the presence of mucus (light blue color indicated by white arrowheads) and subepithelial cells (black arrow) The bronchus was washed before cryo-preservation. ALI: air-liquid interface. Scale bars represent 100 µm.

To overcome limitations in reproducibility, availability, and transferability, the establishment of new human airway tissue models is necessary. Therefore, we generated human 3D airway tissue models using Calu-3 cells. To generate a biologically relevant 3D airway tissue model and to receive proper polarization of the Calu-3 cells, we also included hpF. Two different 3D airway tissue models were compared: a submerged and an ALI model. Calu-3 cells and hpF were co-cultivated on a decellularized porcine small intestinal submucosa without the serosa (Sis-ser) representing a biological collagen scaffold (Figure 1). In submerged 3D models, a monolayered epithelium formed apically within 14 days (Figure 1A).

The hpF migrated basally into the collagen scaffold, and after 14 days, they were found deep within the matrix. Alcian blue staining, used to detect mucus, showed very small areas of light blue staining indicating a modicum of the mucus. In contrast, in ALI models, a multilayered epithelium developed within 7 days after starting the ALI cultivation and progressed over time (Figure 1B, see day 14 and Figure S1 in the Supplemental Material for day 21 and 28). The epithelial layer thickness was 87.79 ± 14.08 µm at day 7 (*n* = 4) and 141.74 ± 21.09 µm at day 14 (*n* = 5).

**Figure 2:**
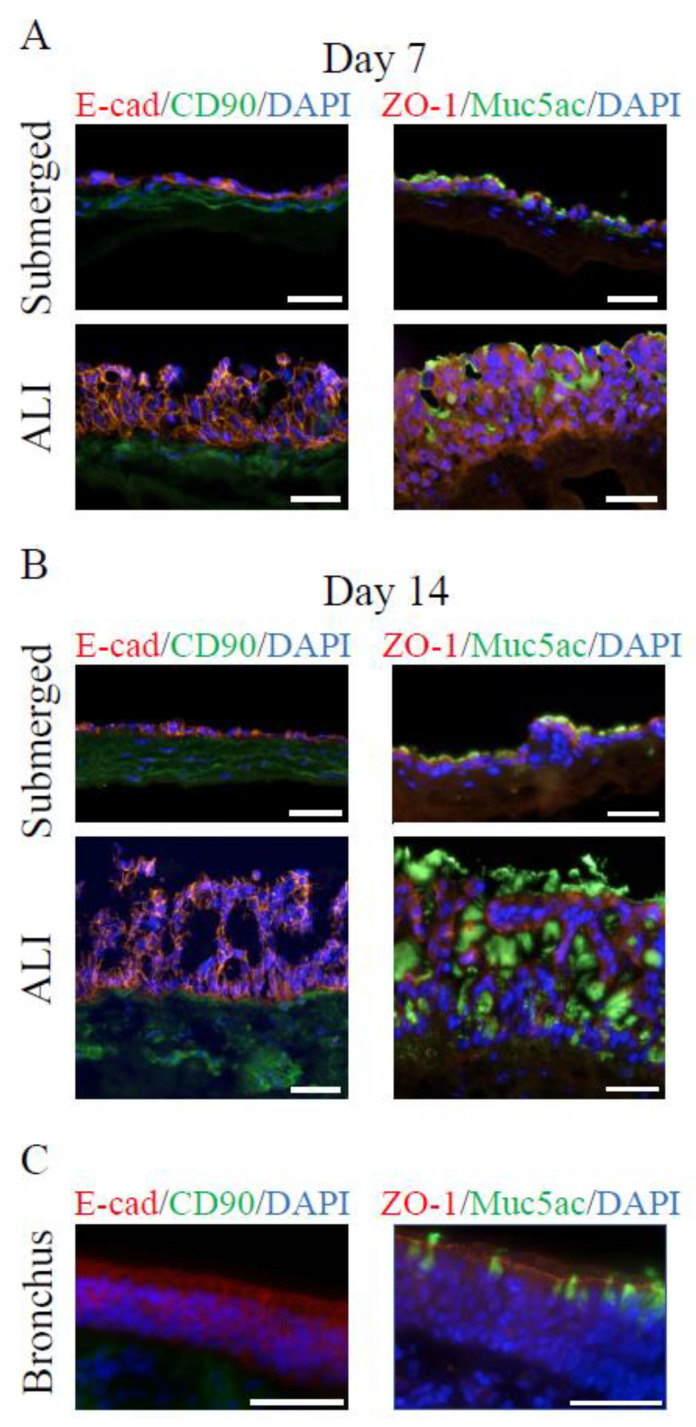
In 3D airway tissue models, tight junctions form, and mucus-producing cells are present. (**A**) Immunofluorescence characterization of 3D submerged and ALI models after 7 days, (**B**) 14 days of cultivation, and (**C**) native bronchial epithelium. Antibody stainings against E-cadherin (E-cad, epithelial cells, red), CD90 (fibroblasts, green), ZO-1 (tight junction marker, red), and Muc5AC (secreted mucus protein, green). Nuclei were stained with DAPI (blue). Three independent experiments were performed. Scale bars represent 50 µm.

**Figure 7:**
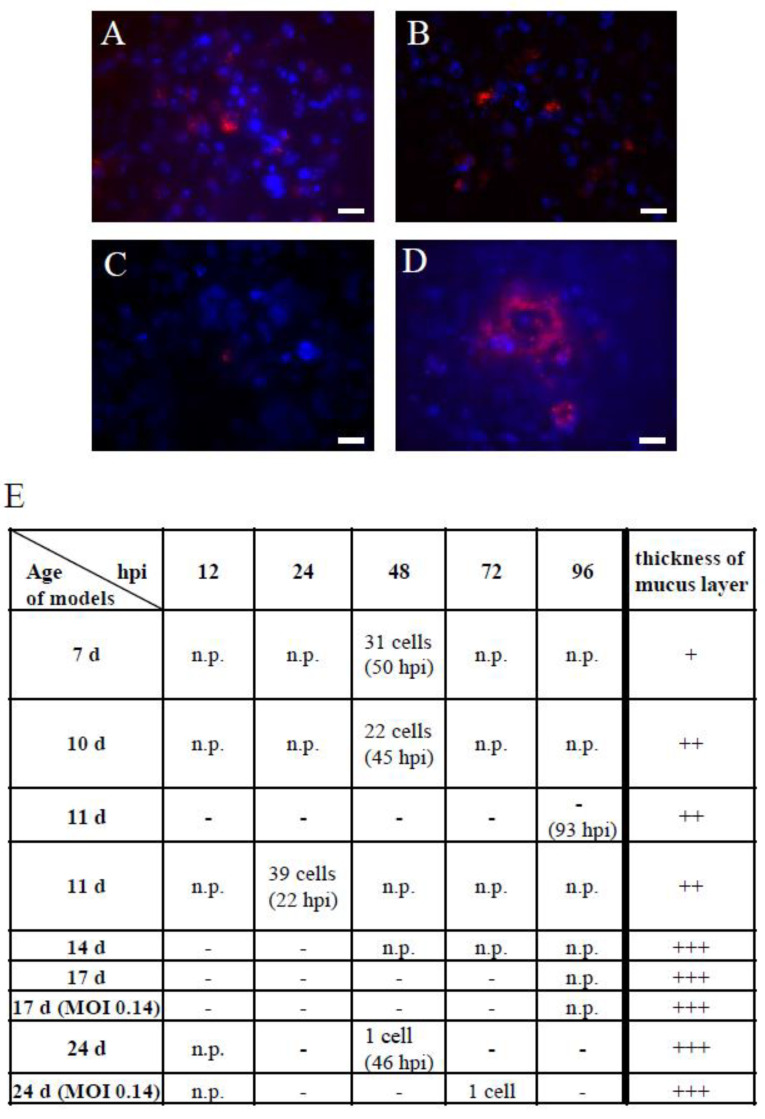
Real-time fluorescence imaging of NS1-RFP IAV infection in 3D ALI models using an MOI of 0.01. (**A**) 7 day old ALI model at 50 hpi, (**B**) 10 day old ALI model at 45 hpi, and (**C**) 24 day old ALI model at 46 hpi showed NS1-RFP fluorescence in detached cells. (**D**) 11 day old ALI model at 22 hpi showing NS1-RFP fluorescence in an attached cell cluster. Scale bars represent 20 µm. (**E**) Overview of the analysis of 3D ALI models with different cultivation times (age of models in days) over a period of 12 to 96 hpi. The models were infected using an MOI of 0.01, with two exceptions using an MOI of 0.14 as noted in the column “age of models”. In the table, the number of NS1-RFP-positive cells found in the entire respective ALI model is indicated. With the increasing age of the models, the thickness of the mucus layer rose, which was histologically estimated from alcian blue stainings of the respective cultivation day (+ = low amount, ++ = medium amount, +++ = high amount). n.p. = not performed, - = no fluorescence observed.

In addition, alcian blue staining demonstrated that ALI models developed mucus-producing cells after 7 days of culture, which was more prominent after 14 days and further increased after 21 and 28 days of ALI cultivation (Figure 1B and Figure S1). The thickness of the mucus layer was estimated histologically using the alcian blue staining (Figure 7E). This staining method indicated an increase in the thickness of the mucus layer with the proceeding age of the 3D ALI model. Comparable with the submerged models, hpF migrated into the collagen scaffold. 3D ALI models at day 7 resembled most of the anatomy of the native bronchial epithelium with mucus present apically (Figure 1C). The airway epithelium is characterized by the expression of markers specific to the different cell types. To further characterize our 3D airway tissue models, immunostainings were performed against an epithelial cell marker present in adherens junctions (E-cadherin), a fibroblast marker (CD90), a tight junction marker (zonula occludens-1 (ZO-1)), and a bronchial specific mucus protein (Muc5AC) produced by goblet cells. In submerged models, Calu-3 cells showed positive immunostaining against E-cadherin at day 7 and day 14 (Figure 2A,B, left) within the monolayer. This suggested that adherens junctions were formed. The bronchial epithelial layer further established tight junctions within 7 days of cultivation (Figure 2A, right), which were maintained in 14 day old 3D submerged models (Figure 2B, right), as confirmed by ZO-1 immunostaining. Next, at day 7 and day 14, a small amount of the bronchial specific mucus protein, Muc5AC, was detected on top of the epithelial layer (Figure 2A,B, right). In ALI models, E-cadherin was found in the entire multilayered epithelium at both stages (Figure 2A,B, left). This was comparable with bronchial epithelial tissue (Figure 2C, left). ZO-1 was found abundantly in Calu-3 cells at day 7 (Figure 2A, right). In 14 day old ALI models, ZO-1 was localized more apically (Figure 2B, right), which was similar to native bronchial epithelial tissue (Figure 2C, right). The presence of the tight junction protein ZO-1 in the 3D tissue models suggested the formation of a functional epithelial barrier. In contrast to the submerged model, in ALI models, the mucus protein Muc5AC was more abundant and the amount increased considerably from day 7 to day 14 (Figure 2A,B, right). In the bronchial epithelium, Muc5AC producing goblet cells could be clearly identified (Figure 2C, right). CD90-positive fibroblasts were present within the collagen matrix in submerged and ALI models (Figure 2A,B, left). In the native bronchial epithelium, a subset of the subepithelial mesenchymal cells was CD90-positive (Figure 2C, left).

To test whether the integration of fibroblasts in our 3D ALI airway model has an impact on the generation of the model, we compared the 3D ALI Calu-3 monoculture with the above described 3D ALI model (Figure S2 in the Supplemental Material). The histological staining demonstrated that in both models, the monoculture as well as the co-culture model, a comparable multilayered epithelium was formed after 7 and 14 days of ALI cultivation. The alcian blue staining showed mucus production in both 3D models at 14 days of ALI cultivation (Figure S2A). Most striking was the presence of the tight junction marker ZO-1. Immunofluorescence staining against ZO-1 in 3D ALI models cultured with and without hpF at 14 days showed less ZO-1 staining in monoculture models than in co-culture models (Figure S2B). These results indicated that the integration of fibroblasts in the 3D ALI airway model had an impact on the formation of tight junctions within the epithelial layer. These results further confirmed the importance of the co-cultivation with fibroblasts for the generation of a relevant 3D airway model.

**Figure 3:**
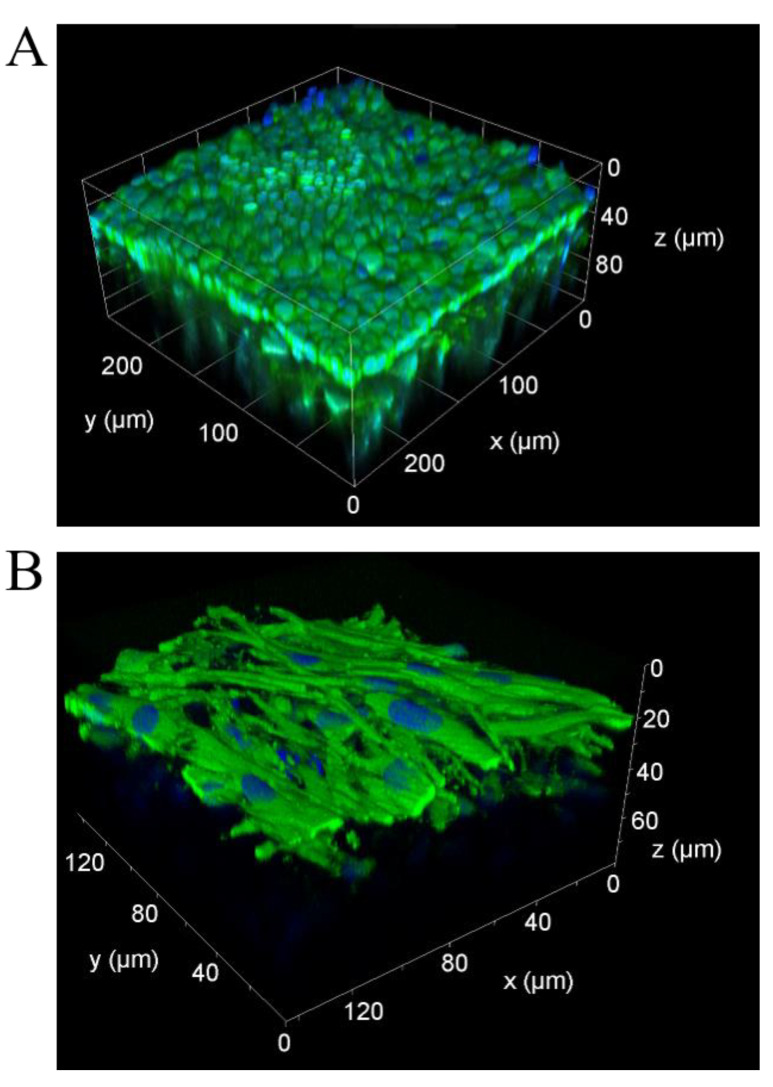
In 3D airway tissue models, tight epithelial layers form and hpF migrate into the collagen scaffold. (**A**) Volume reconstruction of images captured by two-photon microscopy. x, *y*-axis = 250 µm, *z*-axis = 120 µm. (**B**) 3D reconstruction of confocal images taken from the bottom shows hpF in the collagen scaffold. x, *y*-axis = 140 µm, z = 70 µm. Cells were stained with calcein-AM (green) and nuclei with Hoechst 33342 (blue).

Due to the thickness of the 3D model and the density of the collagen scaffold, two-photon and confocal microscopy were performed for anatomical characterization of the model (Figure 3). Both techniques allow imaging of intact living 3D airway tissue models. Calcein-AM and Hoechst 33342 staining were used to define the cell shape and to stain the nuclei, respectively. With two-photon microscopy (Figure 3A), a tissue penetration of 120 µm was reached whereas confocal microscopy allowed for scanning up to 70 µm in depth (Figure 3B). Volume reconstruction of two-photon images illustrated a tightly formed epithelial layer that had a rippled surface (Figure 3A). To visualize fibroblasts more properly, the 3D models were scanned upside down. Confocal microscopy showed that hpF migrated into the collagen scaffold and formed a complex network (Figure 3B).

Our characterization of the 3D airway models showed that in submerged and ALI models an epithelial barrier with tight junctions was formed. The hpF repopulated the scaffold and formed a subepithelial mesenchymal cell layer. In ALI models, a multilayered epithelium with an abundant mucus production was present. Altogether, these features resembled major characteristics of the native bronchial epithelium.

### 3.2. NS1-RFP IAV Replication Is Similar to A/PR/8/34 Wild-Type Virus Strain Replication

**Figure 4:**
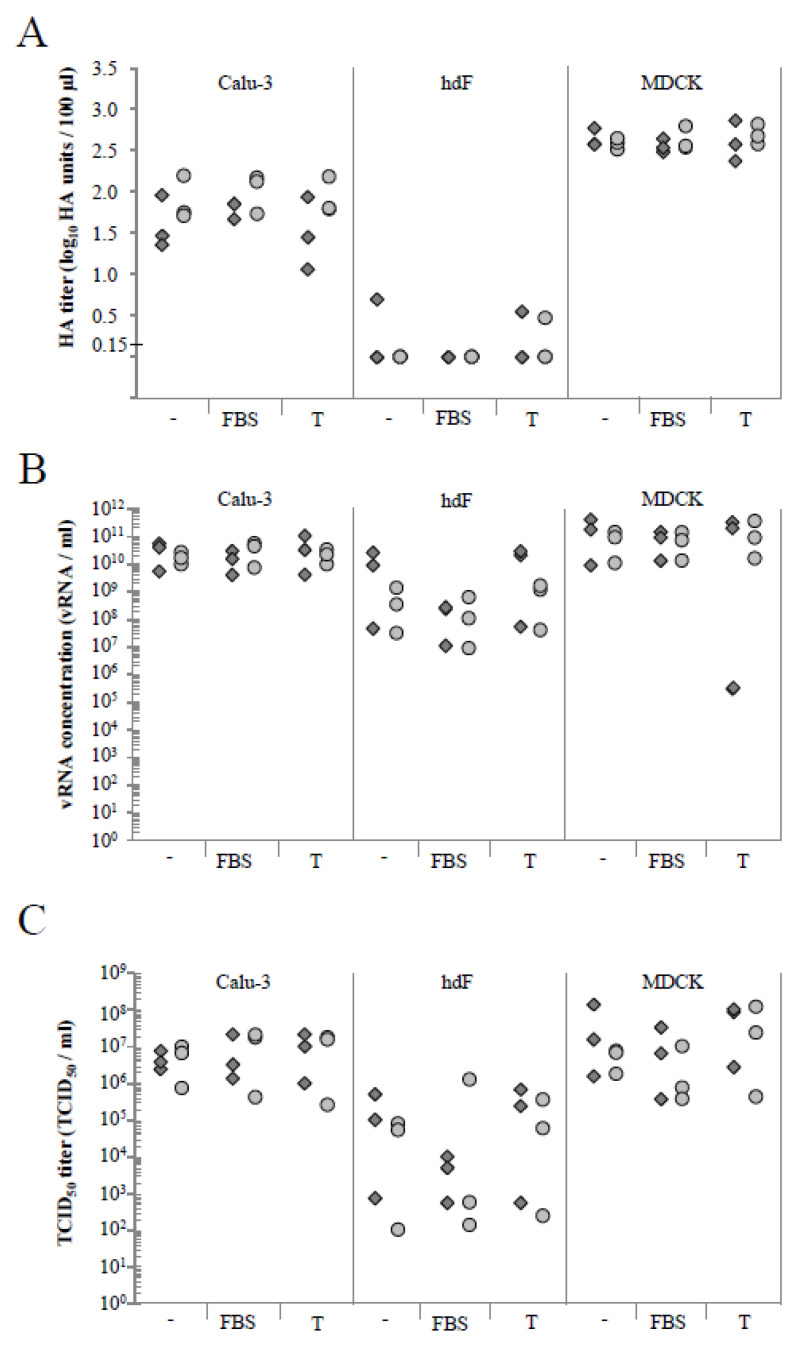
Comparison of virus replication of A/PR/8/34 and NS1-RFP influenza A virus (IAV) in Calu-3 cells, hpF, and MDCK cells under different medium conditions. Data show the respective titer at 24 h post infection (hpi) for Calu-3 cells, hpF, and MDCK cells cultured in two-dimension (2D) (6 well plates) and infected at a multiplicity of infection (MOI) of 1 with A/PR/8/34 or NS1-RFP viruses in basal medium (-), with fetal bovine serum (FBS) or with trypsin (T). (**A**) HA assay (detection limit of 0.15 log_10_ (HA units/100 µL)), (**B**) RT-qPCR, and (**C**) TCID_50_ assay. Shown are the values of each of the three independent experiments. Light grey circle = A/PR/8/34 virus; dark grey square = NS1-RFP virus; hpF = human primary fibroblasts.

The reporter virus NS1-RFP was used, because it allows live-cell imaging [14]. To demonstrate the suitability of this IAV strain for our infection studies, the replication of this strain was compared to the A/PR/8/34 wild-type virus strain (Figure 4). For this purpose, Calu-3 cells, hpF, and MDCK cells acting as positive control were cultured in 6 well culture plates and infected either with the NS1-RFP or the A/PR/8/34 wild-type IAV (both with multiplicity of infection (MOI) of 1). This first experiment served as a scouting experiment for the subsequent real-time microscopy. Therefore, infection under different cell culture conditions were tested. All three different cell types were cultivated without or with fetal bovine serum (FBS), or in the presence of trypsin during infection. FBS was supplemented because it was shown that Calu-3 cells require FBS to maintain a functional epithelium. In ALI cultures, a lack of FBS would also induce the hypersecretion of glycoproteins by Calu-3 cells [24]. Although Calu-3 cells can be infected without exogenous trypsin [2], we wanted to test whether the addition of trypsin would substantially boost IAV infection. The HA titer correlating to the total number of virus particles at 24 h post infection (hpi; log10 (HA units/100 µL)) is shown in Figure 4A. Infection of MDCK cells showed a higher HA titer compared to Calu-3 cells, independent of the culture medium and virus strain (Table S1). In hpF, almost no virus particles could be detected. Infection of Calu-3 cells with the NS1-RFP IAV resulted in slightly lower HA values compared to the A/PR/8/34 wild-type virus strain (Table S1). In contrast, hpF showed slightly higher values for NS1-RFP IAV replication in basal and trypsin supplemented media. Absence of HA activity was found in hpF cultures containing FBS. Very few virus particles could be measured in hpF cultured without FBS or in the presence of trypsin. In all MDCK and Calu-3 cell cultures, the HA titers were similar, irrespective of the applied culture medium (Table S1).

To reassess these data, we performed RT-qPCR analyses as an additional method to determine the concentration of virus particles in the supernatant (Figure 4B). RT-qPCR confirmed the highest values of viral RNA (vRNA) for MDCK cell cultures followed by Calu-3 cells and hpF (Table S2). Infection of Calu-3 cells, hpF, and MDCK cells with the NS1-RFP IAV resulted in slightly higher values than for the A/PR/8/34 wild-type virus strain. Exceptions were Calu-3 cells and hpF cultivated with FBS. Here, the vRNA concentration of NS1-RFP IAV was slightly lower. This was comparable with the data acquired for Calu-3 cells from the HA assay. In contrast to the HA assay, in hpF supernatants, vRNA could be detected. This can be related to the difference in the sensitivity of the two assays.

Next, we measured the concentration of infectious units via the tissue culture infectious dose 50 (TCID_50_) assay (Figure 4C). In general, the same trend as for HA and RT-qPCR data was found, i.e., IAV infection of MDCK cells showed slightly higher TCID_50_/mL values than Calu-3 cells followed by hpF (Table S3). Infections of Calu-3 cells with the NS1-RFP or A/PR/8/34 IAV resulted in similar TCID_50_/mL values for the respective culture conditions. For the FBS-treated cell cultures, slightly lower TCID_50_/mL values were measured for infection with NS1-RFP IAV.

In summary, we found that the NS1-RFP IAV can be utilized to infect Calu-3 cells. The addition of FBS did not interfere in general with the infection of Calu-3 cells and the addition of exogenous trypsin did not substantially enhance the infection.

### 3.3. MOI 0.01 and the Presence of FBS Are Suitable to Infect Calu-3 Cells with the NS1-RFP IAV

One major goal of this work was to microscopically follow the IAV infection in 3D airway models. For this purpose, a suitable NS1-RFP IAV titer that allows us to follow the infection during imaging had to be determined. Therefore, infections of Calu-3 cells using three different MOIs (0.01, 0.1, and 1) in basal medium with and without trypsin or FBS at 9 hpi, 12 hpi, and 24 hpi were compared. In the HA assay, the titers using NS1-RFP or A/PR/8/34 viruses at an MOI of 1 increased over time (Figure S3A in the Supplemental Material). Both viruses showed a comparable but culture medium-specific course of infection. By lowering the MOI to 0.1, the HA titer could only be detected at 24 hpi for both viruses (Figure S3B). For infections at MOI of 0.01 using the NS1-RFP IAV, the HA titer was below the detection limit (0.15 log HA units/100 µL corresponding to about 3 × 10^7^ virions/mL, [19]) at 24 hpi, whereas few A/PR/8/34 wild-type virus particles could be measured at 24 hpi (Figure S3C). RT-qPCR analyses documented increasing vRNA concentrations from 9 hpi to 24 hpi (Figure S4). The vRNA concentration decreased with lower applied MOI as expected. At an MOI of 1 (Figure S4A) and 0.1 (Figure S4B), the vRNA concentration was overall slightly higher after infection with the NS1-RFP IAV compared to the A/PR/8/34 wild-type virus. At an MOI of 0.01, no differences in the vRNA concentration could be observed comparing both virus strains (Figure S4C). Higher vRNA concentrations in supernatants were determined in samples infected in the presence of trypsin. The supplementation with FBS showed no obvious influence on the progress of the infection.

Results of the TCID_50_ assay (Figure S5) supported our RT-qPCR findings: At an MOI of 1, the TCID_50_ of NS1-RFP IAV increased slightly over time (Figure S5A) for all tested conditions, whereas with an MOI of 0.1 the increase was more pronounced (Figure S5B). At the lowest tested MOI of 0.01, the TCID_50_ with the NS1-RFP IAV was in general lower at 24 hpi (Figure S5C). This could indicate that the infectious titer might further increase and sampling at a later time point could have been useful. Again, the presence of FBS did not change the overall dynamics of infection.

In summary, the infection progression of the NS1-RFP IAV virus strain over time was similar to the wild-type virus strain. The use of MOI 0.01 and the addition of FBS were both suitable for infection of Calu-3 cells. Under these conditions, a slow increase in the concentration of infectious virions was achieved that could support real-time imaging of the course of IAV infection in the tissue.

### 3.4. Real-Time Fluorescence Imaging of NS1-RFP IAV Infection in 2D Calu-3 Cell Cultures

**Figure 5:**
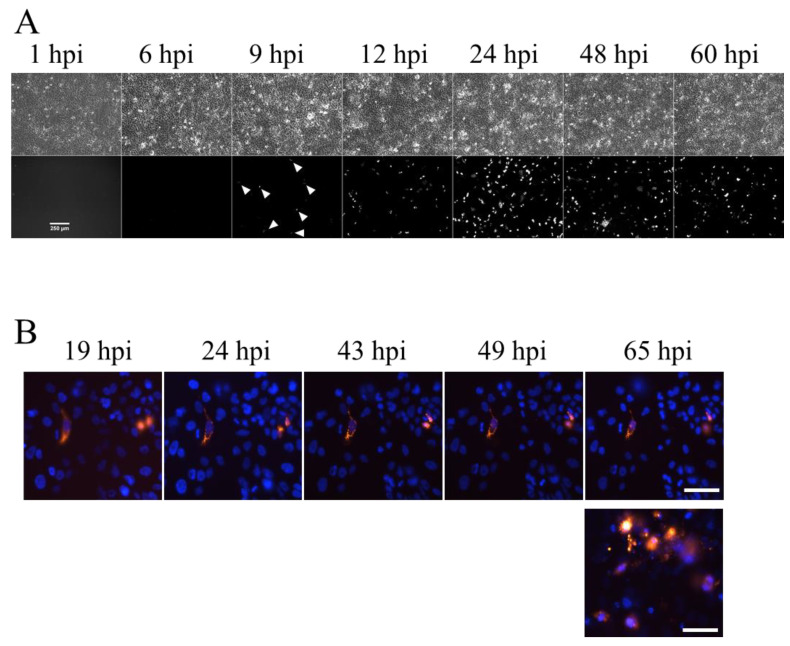
Real-time fluorescence imaging of NS1-RFP IAV infection in 2D Calu-3 cell cultures using MOI 0.01. (**A**) Overview recordings of time-lapse images recorded from 1 hpi to 60 hpi. Arrow heads indicate first RFP-positive cells at 9 hpi. Upper images = transmitted light, lower images = NS1-RFP fluorescence signals. (**B**) A representative example of real-time fluorescence images at a single position during 65 hpi (NS1-RFP signals (red)) shows no changes over time. The image (lower lane, right) presents strong and widespread NS1-RFP signals at 65 hpi within the same cell culture. Experiments were performed three times independently. Nuclei were counterstained with Hoechst 33342 (blue). Scale bars represent 250 µm (**A**) and 25 µm (**B**), respectively.

To establish real-time fluorescence imaging, first, 2D Calu-3 cell cultures were infected with the NS1-RFP IAV at an MOI of 0.01 in a culture medium containing FBS and monitored for 65 hpi via the intracellular virus fusion protein NS1-RFP (Figure 5). These experiments should give us information about the properties of the red fluorescence signal and the progression of infection in 2D cell cultures. In general, an increase of infected NS1-RFP-positive cells over time was observed (Figure 5A). At early infection time points (1 and 6 hpi), no NS1-RFP signal was visible. The first fluorescence signals in single cells were detectable at 9 hpi. After that the number of infected cells (NS1-RFP-positive cells) increased. The fluorescence signal in the cells remained stable until at least 65 hpi (Figure 5B). In addition to the infected cells that were still attached at the surface of the cell culture dish, the majority of the NS1-RFP-positive cells detached (Figure 5B, lower image, right). This finding may indicate a normal replication cycle at low MOI with cell lysis due to virus-induced apoptosis at later time points (>24 hpi). Quantification via the HA assay was performed only at 48 and 60 hpi (Figure S6 in the Supplemental Material), as IAV particles in the supernatant are rather stable. On the other hand, the more sensitive RT-qPCR and TCID_50_ assays allowed for the quantifying of virus particles already at 6 hpi. The vRNA concentration increased from 1.82 × 10^6^ vRNA/mL (6 hpi) to 1.29 × 10^10^ vRNA/mL (60 hpi) and the TCID_50_ increased from 4.71 × 10^2^ TCID_50_/mL (6 hpi) to 5.14 × 10^5^ TCID_50_/mL (60 hpi). Starting from 12 hpi, the concentration of vRNA increased constantly, while the concentration of infectious particles reached a plateau (Figure S6). To further analyze the spreading of infection in more detail, cell cultures were microscopically monitored at four to seven separate positions over time (Figure 5B, upper row). These images show the course of infection at one individual position from 19 to 65 hpi. During the entire observation time, no spreading to directly adjacent neighboring cells was observed within the microscopically monitored individual positions. However, the infection of cells progressed overall, and at 65 hpi, infected NS1-RFP-positive cells were eventually distributed evenly (Figure 5B, lower image, right).

### 3.5. Real-Time Fluorescence Imaging of Infection in Submerged 3D Airway Models Allows Monitoring of Infection Progression

**Figure 6:**
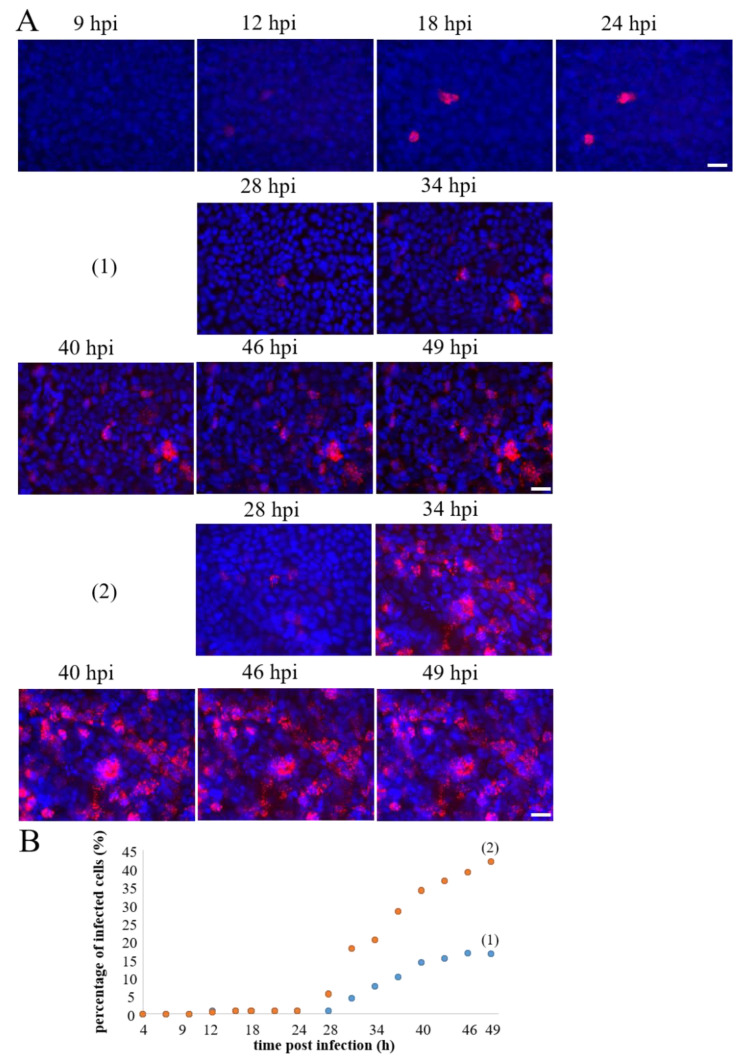
Real-time fluorescence imaging of NS1-RFP IAV infection in 3D submerged models using an MOI of 0.01. (**A**) Representative examples of time-lapse sequences were recorded between 9 to 24 hpi and 28 to 49 hpi, respectively. These models were pre-cultivated for 18 days and showed the first NS1-RFP fluorescence between 9 and 12 hpi. From 28 hpi onwards, the percentage of NS1-RFP-positive cells gradually increased (example (1) and (2)). (**B**) Quantification of the percentage of infected cells of these positions was between 4 and 49 hpi. The percentage of NS1-RFP-positive cells was calculated by cell counting of the total cell number (Hoechst-positive nuclei) and the NS1-RFP-positive cells in the respective images. Scale bars represent 25 µm.

In order to establish real-time fluorescence imaging of IAV infection in the 3D airway model, first, infections in submerged 3D airway models were performed (Figure 6). We used submerged models cultivated for 13 to 24 days and infected them apically in basal medium containing FBS with MOI of 0.01 or 1. After removing the residual virus, the infection progression was followed via fluorescence microcopy of the NS1-RFP between 4 and 49 hpi (overview in Table 1).

All analyzed submerged 3D models could be infected irrespective of the age of the model. The first fluorescence signals appeared between 9 and 12 hpi (Figure 6A, Video S1 in the Supplemental Material), which was comparable with our observations from 2D cell cultures. The amount of NS1-RFP-positive cells at the analyzed positions remained stable between 9 and 24 hpi, exemplarily shown for one position (Figure 6A,B). In all 3D submerged models, with one exception, the overall number of NS1-RFP-positive cells increased considerably after 24 hpi until 49 hpi as detected by real-time imaging (Table 1). The infection progression, which was monitored for different positions within the 3D airway models, however, varied between the positions. In an 18 day old model shown here, at one position (1), 16.5% of the cells were NS1-RFP-positive at 49 hpi. Another position (2), however, showed an increase of NS1-RFP-positive cells up to 42% (Figure 6A,B). Video S2 illustrates the infection progression at this position between 24 and 49 hpi. Again, in the other positions, no newly infected NS1-RFP-positive cells were observed, which was similar to the observations in 2D Calu-3 cell cultures. The infection progression in 3D submerged models, therefore, exhibited a clear difference compared to the infection of 2D Calu-3 cell culture, namely that infection progression in the immediate vicinity of infected cells could be observed. Live-cell imaging of 3D submerged models further showed propagation of NS1-RFP starting from infected cells (Video S3). Interestingly, in one case, we observed that dividing Calu-3 cells transmitted NS1-RFP (Figure S7). Infections of the subepithelial layer were not found, and likewise, in 3D hpF monoculture models no NS1-RFP signals could be detected (data not shown). In summary, the 3D submerged model could be infected with the NS1-RFP IAV at a low MOI. The infection progression could be studied over at least 49 hpi. This 3D airway model thus showed a replication dynamic that could be monitored in real time via microscopy.

### 3.6. Real-Time Fluorescence Imaging of Infection in ALI Airway Models Showed a Slower Progression of Infection

In comparison to the submerged 3D model, the ALI model displayed a mucus barrier whose thickness depended on the cultivation time. This model therefore possessed one more feature of bronchial epithelial tissue. Therefore, we were also interested to perform real-time imaging of NS1-RFP IAV infection in the 3D ALI model (Figure 7). 

Depending on the advancing age of the ALI model, the thickness of the epithelial and the mucus layer increased (Figure 1, Figure 7E and Figure S1). Due to these age dependent differences, we used 3D ALI models that were differentiated for 7 to 24 days. In contrast to the general practice (e.g., [25]), we did not remove the produced mucus before infection and during infection progression, because it is known that mucus can display a natural infection barrier. For the IAV infection, we used a low MOI of 0.01 or 0.14, which is comparable with the infections in submerged 3D models. Due to the multilayered structure and the intense mucus production, the fluorescence microscopy of this model posed a challenge. In “younger” 3D ALI models (7–10 days old) between 45 and 50 hpi, several detached NS1-RFP-positive cells were found (Figure 7A,B,E). In an 11 day old ALI model at 22 hpi, a larger NS1-RFP-positive cluster of cells could be observed (Figure 7D,E). In “older” models (14–24 days old) which were strongly covered with mucus, only single NS1-RFP-positive cells could be detected at 46 hpi and 72 hpi, respectively (Figure 7C,E). These findings indicated that our ALI model featured a functional mucus barrier in addition to the epithelial barrier. Using the same low MOI for infections as used for submerged models, the data suggested a slower progression of infection in ALI models. However, it should also be considered that “older” ALI models (older than 14 days) are more difficult to analyze with microscopy. Hence, further improvement concerning the microscopy technique used is needed. In conclusion, we could establish NS1-RFP IAV infections in ALI models. We further demonstrated that the ALI model is suitable to analyze the impact of the epithelial and most of all the mucus barrier of a respiratory virus infection.

## 4. Discussion

Complex 3D airway tissue models are an alternative to animal experiments to study respiratory virus infection. Real-time imaging of the virus spreading at a cellular level makes specific demands on the 3D airway model. Lung organoids, a well-established 3D model, have to be sheared mechanically for infection to expose the apical surface. The imaging of the infection spreading from cell to cell in these organoids is difficult to realize due to their anatomy. 3D airway models composed of the primary airway epithelium also have limitations due to their donor variability (low reproducibility), slow and limited growth properties, and long differentiation time (limited availability). In addition, the existing 3D models often consist of the epithelial layer only and lack the ECM including the basement membrane and the stromal cellular component that are required for a biologically relevant 3D airway model [4]. For these reasons, a need of new in vitro airway models exists. The use of established airway epithelial cell lines could overcome these limitations. Hence, we tested whether 3D airway models composed of Calu-3 cells and hpFs on a collagen scaffold could be used for monitoring respiratory virus spreading in vitro. In this study, we have established 3D submerged and ALI airway tissue models composed of airway epithelial cells and fibroblasts. In both 3D models, a functional epithelial barrier due to the presence of fibroblasts developed. Although Calu-3 cells are known to form tight junctions in 2D and 3D monocultures (e.g., [24,26,27]), we and others have shown that there is less ZO-1 protein present compared to cultures of normal bronchial epithelial cells [12] and to Calu-3/fibroblast 3D ALI co-cultures [28]. The use of standard inserts (e.g., with polyethylene terephthalate (PET) membrane) has also been shown to negatively influence the ZO-1 expression compared to 3D ALI models on collagen-hyaluronate scaffolds [28]. These findings further support the importance of fibroblasts and the scaffold properties in 3D airway models for the formation of an epithelial barrier.

Additionally, mucus was secreted, forming a mucus barrier. Both characteristics are highly relevant in the defense against viral pathogens [29]. Furthermore, in 3D submerged models, a monolayered epithelium formed, but in ALI models a multilayered epithelium with reproducible epithelial layer thickness and anatomy developed which resembled the native bronchial epithelium. In our hands, Calu-3 cells grew rapidly and consistently. They can also be used over a wide range of passage numbers [30].

Human fibroblasts, even though they are derived from primary cells, are robust and proliferative cells over several passages. A former study demonstrated that there was no appreciable donor-to-donor variability using different fibroblast donors in 3D tissue models [31], therefore this component of the model could be kept constant without impairing the biological relevance of the 3D models. Accordingly, we were able to generate 3D models that are standardized, fast-to-generate, and reproducible. Thereby, these models are convenient to establish real-time imaging of respiratory virus spreading.

To follow virus spreading in vitro, we used a genetically modified influenza NS1-RFP virus strain [14]. The RT-qPCR and TCID_50_ assays, used to establish the infection conditions, were suitable to follow infection and virus production in 2D cell cultures even at an MOI of 0.01, whereas the HA assay was not sensitive enough to monitor early increases in the virus titer. Our analysis of virus replication thus showed that the NS1-RFP virus behaved comparably to the influenza A/PR/8/34 wild-type virus strain. The addition of trypsin was not mandatory and did not further boost the infection of Calu-3 cells. The addition of FBS did not influence the concentration of infectious virions. In 2D hpF cultures, very low concentrations of vRNA and infectious virions could be detected. In the presence of FBS, these concentrations were even lower but still detectable. This may indicate that hpF cultured in 2D are susceptible to IAV infection. In 3D airway models, however, infection of hpF using an MOI of 0.01 could not be visually observed. This could suggest that in our 3D models, hpF were protected from the IAV either due to the presence of the mucus barrier, the epithelial layer, or the surrounding extracellular matrix.

We first, tested the real-time monitoring of IAV infections in 2D Calu-3 monolayer cultures. There, only infections of single cells were found, whereas neighboring cells stayed uninfected. However, in general, the number of infected cells increased over time. The reason for this may be that in our 2D Calu-3 cell cultures, virus particles were mainly released into the medium and infected other cells randomly.

Next, we monitored IAV infections in 3D airway tissue models. Interestingly, in the submerged 3D airway model, we observed different courses of IAV infection spreading in parallel. Besides single-cell infections, we found evidence for cell-to-cell transfer of virus proteins, which suggested different mechanisms to be involved in contrast to the infection in 2D cultures. We also observed the formation of infected clusters of cells, which was described very recently for lung organoid cultures as well [32].

The 3D ALI model developed a distinct mucus layer. In our study, we did not remove the mucus before infection on purpose, because we wanted to generate 3D models that would more closely mimic the in vivo situation. In these airway tissue models, the progression of infection was remarkably slower under the same infection conditions as used for the 3D submerged model. This let us conclude that progression of infection was reduced most likely due to increased apical mucus secretion. Therefore, in future experiments, a prolonged observation time for the real-time microcopy should be considered.

We realized that the analysis of 3D ALI models using microscopy techniques can be challenging because of the thickness of the epithelial layer and the mucus barrier. To overcome this difficulty, we used objectives with long working distances. Alternatively, two-photon microcopy can be used to further increase optical penetration depth.

Furthermore, we observed a spatial heterogeneity of infections in our 3D airway models. One could speculate that the NS1-RFP virus lost its RFP sequence. Manicassamy and colleagues observed that nearly 70% of the NS1-GFP virus population in the lungs of infected mice remained GFP-positive (day 6) [1]. Therefore, loss of the RFP sequence does not seem to be highly relevant for our experiments either, considering the comparatively short observation periods of 49 hpi (submerged) and 96 hpi (ALI), respectively. As described earlier, spatial heterogeneity could also be caused by the natural heterogeneity of factors involved in virus infection and spreading [33]. It is conceivable that the heterogeneity in our 3D models reflects this physiological situation. Nevertheless, the microscopic analysis and its quantification turned out to be more complicated. These issues are currently difficult to solve and should be addressed in the future.

The primary innate defense mechanism of the bronchus also involves cilia movement. In our 3D airway model, Calu-3 cells did not form cilia under our ALI culture conditions, as also reported earlier [34]. To study the impact of cilia movement on virus infection, another cell line like HBEC3-KT could be utilized [34]. Alternatively, 3D models composed of primary airway epithelial cells with fibroblasts could be used, but only for proof of principle experiments due to the already mentioned limitations.

After setting up the presented model system, the next step would be to evaluate more influenza virus strains and to compare them against this reference virus.

## 5. Conclusions

Our findings suggest that our 3D airway models are biologically relevant for studies of virus spreading due to the observed differences in infection spreading compared with 2D cultures. An advantage of these 3D models is that they can be generated rapidly and reproducibly. This makes them applicable for a variety of studies on virus infection mechanisms next to other 3D airway models and animal studies.

## Figures and Tables

**Table 1:** Summary of infection experiments in submerged models.

			Hours Post Infection (hpi)						
Age of Model (Days)	MOI	N	4	9	12	24	28	37	49
13	0.01	1	n.p.	n.p.	n.p.	n.p.	++ (27 hpi)	+++	n.p.
13	1	1	n.p.	n.p.	n.p.	++	n.p.	n.p.	n.p.
14	0.01	2	-	+	+	+	+	+	+ (43 hpi)
14	0.01	1	n.p.	n.p.	n.p.	n.p.	n.p.	n.p.	+++ (48 hpi)
18	0.01	2	-	-	+	+	+	++	+++ (49.75 hpi)
21	0.01	2	-	-	+ (14 hpi)	n.p.	n.p.	n.p.	++
24	0.01	1	-	-	+ (11.5 hpi)	+ (22 hpi)	++ (32 hpi)	n.p.	n.p.
24	1	1	n.p.	n.p.	n.p.	+ (22 hpi)	++ (32 hpi)	++	+++

Submerged 3D models with the cultivation stage (age of model in days) stated were infected with the NS1-RFP IAV using the specified MOI. The models were analyzed via fluorescence microscopy over the time frame of 4 to 49 hpi at several positions each. The observed amount of NS1-RFP-positive cells at several positions are defined as follows:”-“: no RFP-positive cells, “+”: one or two RFP-positive cells, “++”: more than two RFP-positive cells, “+++”: more than ~15% RFP-positive cells, n.p.: not performed, N: number of analyzed 3D models.

## Data Availability

Not applicable.

## Supplementary Materials

The following supporting information can be downloaded at: https://www.mdpi.com/article/10.3390/cells11223634/s1. Table S1: HA assay of NS1-RFP or A/PR/8/34 virus strain infected Calu-3 cells, hpF, and MDCK cells at 24 hpi (MOI of 1, seed virus MDCK cell adapted); Table S2: RT-qPCR of the supernatant of NS1-RFP or A/PR/8/34 virus strain infected Calu-3 cells, hpF, and MDCK cells at 24 hpi (MOI of 1, seed virus MDCK cell adapted); Table S3: TCID_50_ assay of NS1-RFP or A/PR/8/34 virus strain infected Calu-3 cells, hpF, and MDCK cells at 24 hpi (MOI of 1, seed virus MDCK cell adapted); Figure S1: Alcian blue staining of 3D ALI models at later cultivation stages; Figure S2 Comparison of 3D Calu-3 monoculture with 3D Calu-3/hpF co-culture models; Figure S3: Comparison of the concentration of total virus particles of A/PR/8/34 and NS1-RFP IAV at different MOI in 2D Calu-3 cell cultures; Figure S4: Comparison of the viral RNA concentration of A/PR/8/34 and NS1-RFP IAV at different MOIs in 2D Calu-3 cell cultures; Figure S5: Comparison of the concentration of infectious particles of A/PR/8/34 and NS1-RFP IAV at different MOIs in 2D Calu-3 cell cultures; Figure S6: Determination of the virus titer found in the supernatant of 2D Calu-3 cell infections with NS1-RFP virus over 60 hpi; Figure S7: Infection of the NS1-RFP virus in 3D submerged model (21 days of cultivation); Video S1: Time-course of NS1-RFP IAV infection between 4 h and 18 h 15 min post infection, in a 18 days old submerged 3D airway model infected with MOI 0.01; Video S2: Time-course of NS1-RFP IAV infection between 28 h and 49 h 45 min post infection, in a 18 days old submerged 3D airway model infected with MOI 0.01; Video S3: Movement of NS1-RFP starting from infected cells between 28 h and 49 h 45 min post infection, in a 18 days old submerged 3D airway model infected with MOI 0.01.
