# Supplementary material for: Human 3D Airway Tissue Models for Real-Time Microscopy: Visualizing Respiratory Virus Spreading
Source: Cells. 2022 Nov 16;11(22):3634. doi: 10.3390/cells11223634 (PMC9688616; doi:10.3390/cells11223634)
Supplement: Supplementary file 1 [file cells-11-03634-s001.zip › Supplemental material.pdf]

Supplemental material

Table S1:

HA assay of NS1-RFP or A/PR/8/34 virus strain infected Calu-3 cells, hpF, and MDCK cells at 24 hpi (MOI of 1, seed virus MDCK cell adapted). Values represent the arithmetic mean with standard deviation of three independent experiments in $\log_{10}$ (HA units/100 µl). n.d. = not detectable (below detection limit of 0.15 $\log_{10}$ (HA units/100 µl)

		basal medium	with FBS	with trypsin
Calu-3	NS1-RFP	1.6 ± 0.32	1.79 ± 0.1	1.48 ± 0.44
	A/PR/8/34	1.88 ± 0.27	2.01 ± 0.24	1.92 ± 0.22
hpF	NS1-RFP	0.7 (single value)	n.d.	0.55 (single value)
	A/PR/8/34	n.d.	n.d.	0.47 (single value)
MDCK	NS1-RFP	2.64 ± 0.11	2.55 ± 0.08	2.6 ± 0.25
	A/PR/8/34	2.61 ± 0.07	2.65 ± 0.14	2.71 ± 0.12

Table S2:

RT-qPCR of the supernatant of NS1-RFP or A/PR/8/34 virus strain infected Calu-3 cells, hpF, and MDCK cells at 24 hpi (MOI of 1, seed virus MDCK cell adapted). Values represent the arithmetic mean with standard deviation of three independent experiments as concentration of viral RNA in vRNA/ml.

		basal medium	with FBS	with trypsin
Calu-3	NS1-RFP	3.28E+10 ± 2.49E+10	1.57E+10 ± 1.24E+10	4.55E+10 ± 5.00E+10
	A/PR/8/34	1.82E+10 ± 8.93E+09	3.69E+10 ± 2.61E+10	2.23E+10 ± 1.22E+10
hpF	NS1-RFP	1.17E+10 ± 1.33E+10	1.74E+08 ± 1.42E+08	1.66E+10 ± 1.49E+10
	A/PR/8/34	6.09E+08 ± 7.30E+08	2.74E+08 ± 3.68E+08	9.60E+08 ± 8.34E+08
MDCK	NS1-RFP	2.25E+11 ± 2.28E+11	8.58E+10 ± 6.89E+10	1.98E+11 ± 1.86E+11
	A/PR/8/34	8.47E+10 ± 6.90E+10	7.75E+10 ± 6.52E+10	1.59E+11 ± 1.86E+11

Table S3:

$TCID_{50}$ assay of NS1-RFP or A/PR/8/34 virus strain infected Calu-3 cells, hpF, and MDCK cells at 24 hpi (MOI of 1, seed virus MDCK cell adapted). Values represent the arithmetic mean with standard deviation of three independent experiments as concentration of infectious virions in $TCID_{50}$/ml.

		basal medium	with FBS	with trypsin
Calu-3	NS1-RFP	4.54E+06 ± 2.65E+06	8.53E+06 ± 1.09E+07	1.07E+07 ± 1.01E+07
	A/PR/8/34	5.81E+06 ± 4.69E+06	1.31E+07 ± 1.11E+07	1.13E+07 ± 9.60E+06
hpF	NS1-RFP	2.00E+05 ± 2.64E+05	5.19E+03 ± 4.72E+03	3.02E+05 ± 3.38E+05
	A/PR/8/34	4.17E+04 ± 3.81E+04	3.97E+05 ± 6.87E+05	1.31E+05 ± 1.80E+05
MDCK	NS1-RFP	5.92E+07 ± 8.59E+07	1.51E+07 ± 1.97E+07	7.41E+07 ± 6.21E+07
	A/PR/8/34	5.32E+06 ± 3.09E+06	3.71E+06 ± 5.45E+06	4.77E+07 ± 6.28E+07

Supplemental Figure Legends:

Figure S1 Alcian blue staining of 3D ALI models at later cultivation stages. In ALI models with older age (day 21 (left) and day 28 (right)), the amount of produced mucus (light blue color, indicated by the white arrowheads) increased. Scale bars represent 100 µm.

Figure S2 Comparison of 3D Calu-3 monoculture with 3D Calu-3 / hpF co-culture models. (A) Alcian blue stainings of 3D ALI models at 7 and 14 days of cultivation, respectively. The upper row shows stainings of 3D ALI Calu-3 monoculture models. The lower row shows stainings of 3D ALI co-culture models. The white arrowheads point at the mucus. (B) Immunofluorescence

stainings against ZO-1 (red) in 3D ALI Calu-3 monoculture (upper row) and Calu-3 / hpF (A) and 50 µm (B), respectively.

Figure S3 Comparison of the concentration of total virus particles of A/PR/8 /34 and NS1-RFP IAV at different MOI in 2D Calu-3 cell cultures. HA assay performed at 9, 12, and 24 hpi with MOI of 1 (A), MOI of 0.1 (B), and MOI of 0.01 (C) with NS1-RFP (left) or A/PR/8/34 IAV (right) in basal medium (dark grey, lozenge), basal medium with FBS (grey, square), and basal medium with trypsin (light grey, triangle). Shown are mean values and standard deviations of five independent experiments.

Figure S4 Comparison of the viral RNA concentration of A/PR/8/34 and NS1-RFP IAV at different MOIs in 2D Calu-3 cell cultures. RT-qPCR performed at 9, 12, and 24 hpi with MOI of 1 (A), MOI of 0.1 (B), and MOI of 0.01 (C) with NS1-RFP (left) or A/PR/8/34 IAV (right) in basal medium (dark grey, lozenge), basal medium with FBS (grey, square), and basal medium with trypsin (light grey, triangle). Shown are the mean values and standard deviations of five independent experiments.

Figure S5 Comparison of the concentration of infectious particles of A/PR/8/34 and NS1-RFP IAV at different MOIs in 2D Calu-3 cell cultures. $TCID_{50}$ assay performed at 9, 12, and 24 hpi with MOI of 1 (A), MOI of 0.1 (B), and MOI of 0.01 (C) with NS1-RFP (left) or A/PR/8/34 IAV (right) in basal medium (dark grey, lozenge), basal medium with FBS (grey, square), and basal medium with trypsin (light grey, triangle). Shown are the mean values and standard deviations of five independent experiments.

Figure S6 Determination of the virus titer found in the supernatant of 2D Calu-3 cell infections with NS1-RFP virus over 60 hpi. The Calu-3 cells were cultured in basal medium with FBS in 6 well plates and infected with MOI 0.01. HA assay in $TCID_{50}$/ml (blue), RT-qPCR in vRNA/ml (grey) and $TCID_{50}$ assay in $TCID_{50}$/ml (orange). Shown are mean values and standard deviations of three independent experiments. The virus standards of A/PR/8/34 (n=3) and NS1-RFP IVA (n=2) served as PCR (grey) and $TCID_{50}$ (orange) control (on the right side, as separated graph).

Figure S7 Infection of the NS1-RFP virus in 3D submerged model (21 days of cultivation). The image was recorded at 48 hpi. In this case, dividing Calu-3 cells with NS1-RFP fluorescence (white arrowhead) were observed. Nuclei were counterstained with Hoechst 33342 (blue). Scale bar represents 25 µm.

Movie Legends:

Video S1 Time-course of NS1-RFP IAV infection between 4 h and 18 h 15 min post infection, in a 18 days old submerged 3D airway model infected with MOI 0.01. First NS1-RFP fluorescence signals were visible at around 12 hpi. Scale bar represent 20 µm.

Video S2 Time-course of NS1-RFP IAV infection between 28 h and 49 h 45 min post infection, in a 18 days old submerged 3D airway model infected with MOI 0.01. Infections progressed in the immediate vicinity over time. Scale bar represent 20 µm.

Video S3 Movement of NS1-RFP starting from infected cells between 28 h and 49 h 45 min post infection, in a 18 days old submerged 3D airway model infected with MOI 0.01. Scale bar represent 20 µm.
