# Supplementary figures and images for: Human 3D Airway Tissue Models for Real-Time Microscopy: Visualizing Respiratory Virus Spreading
Source: Cells. 2022 Nov 16;11(22):3634. doi: 10.3390/cells11223634 (PMC9688616; doi:10.3390/cells11223634)

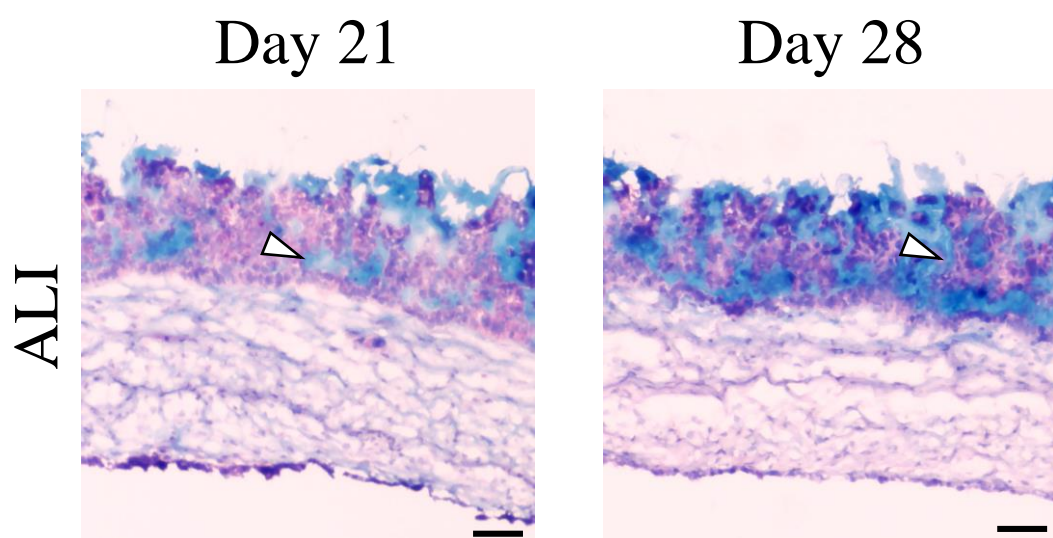

Figure S1

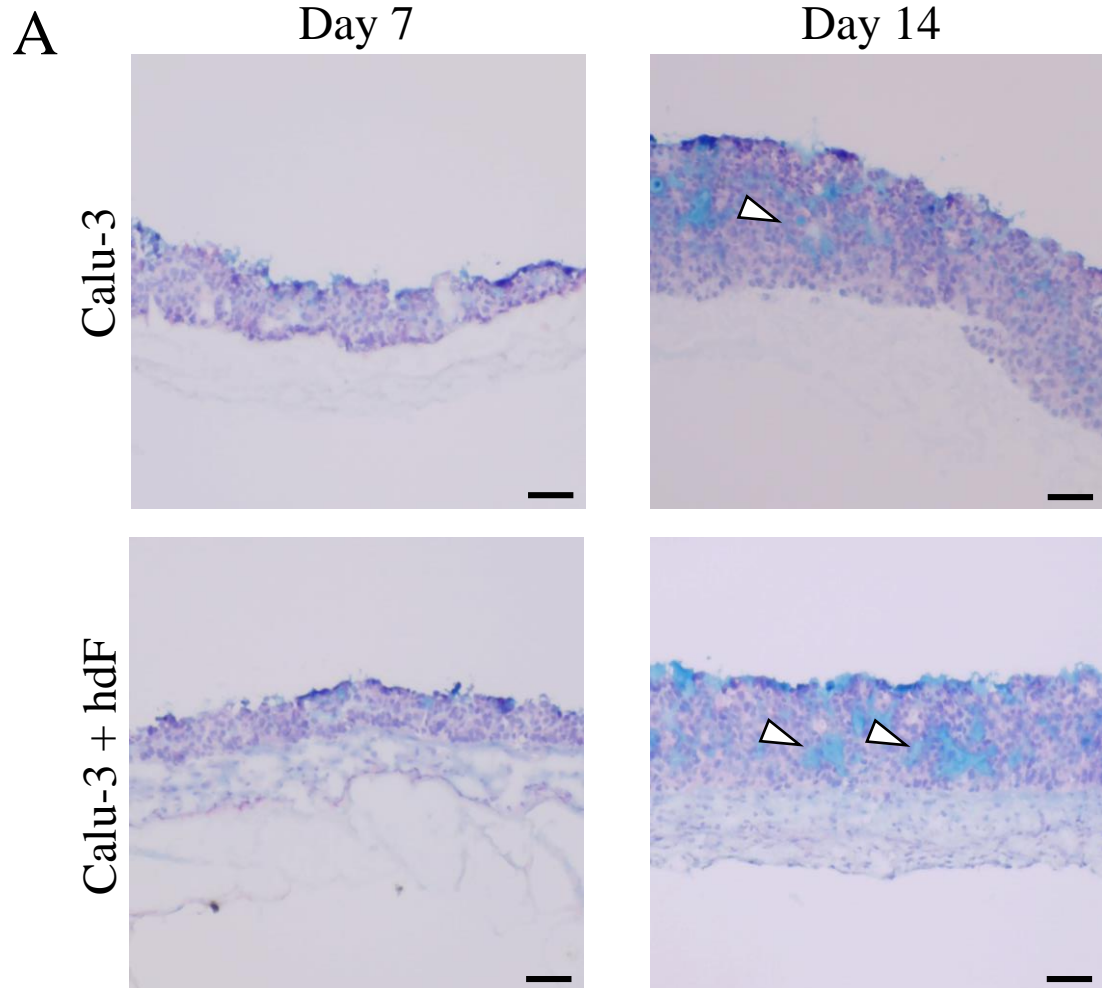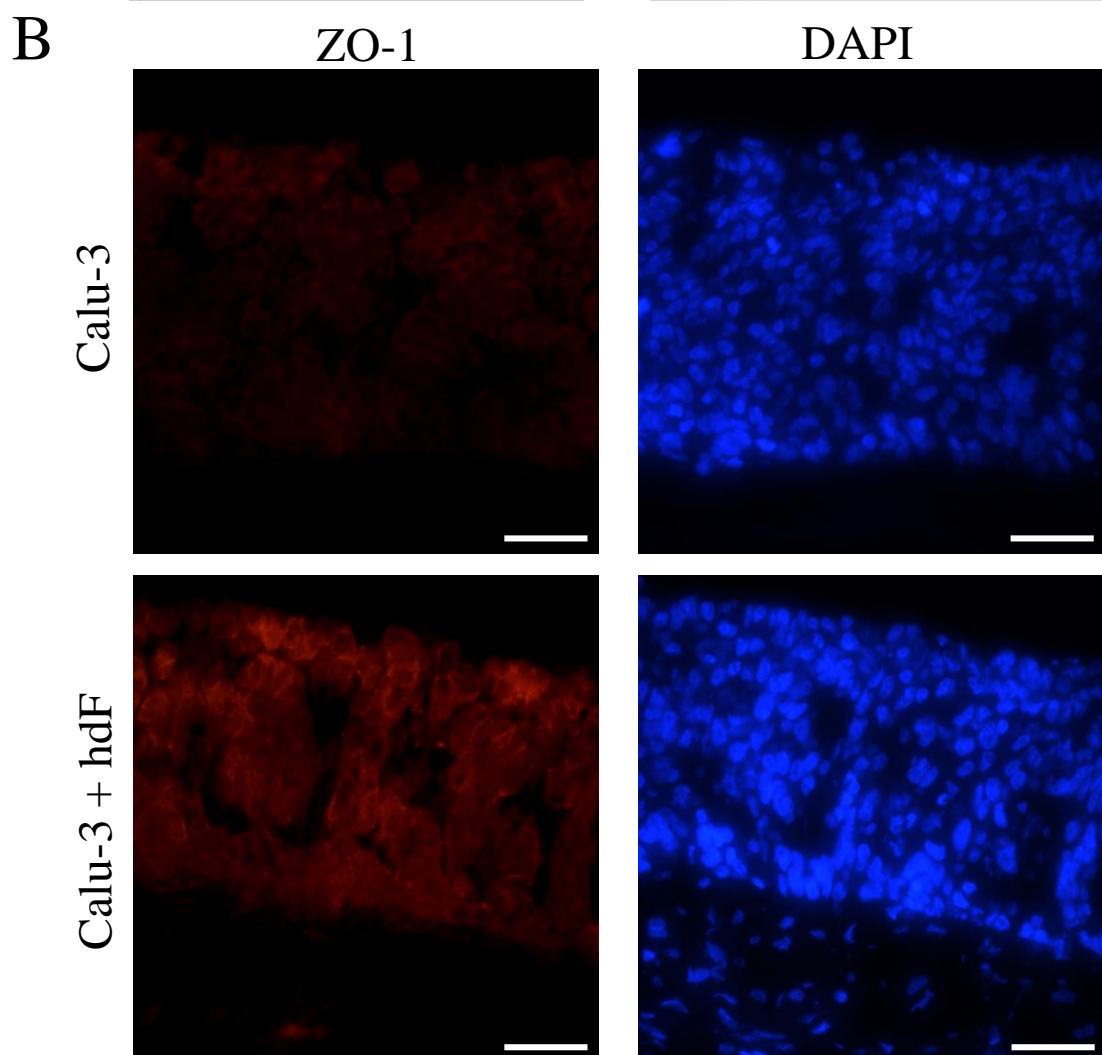

Figure S2

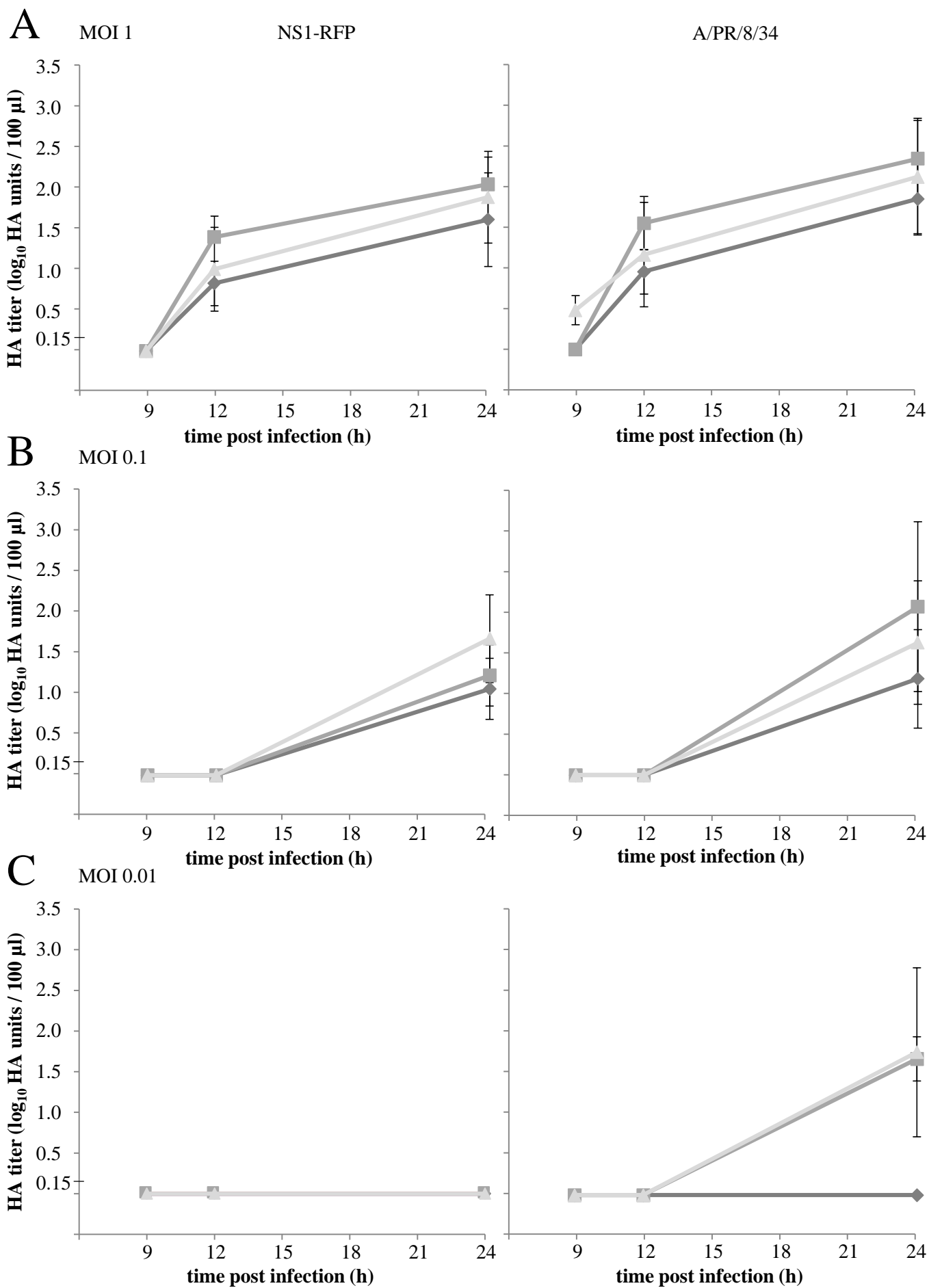

Figure S3

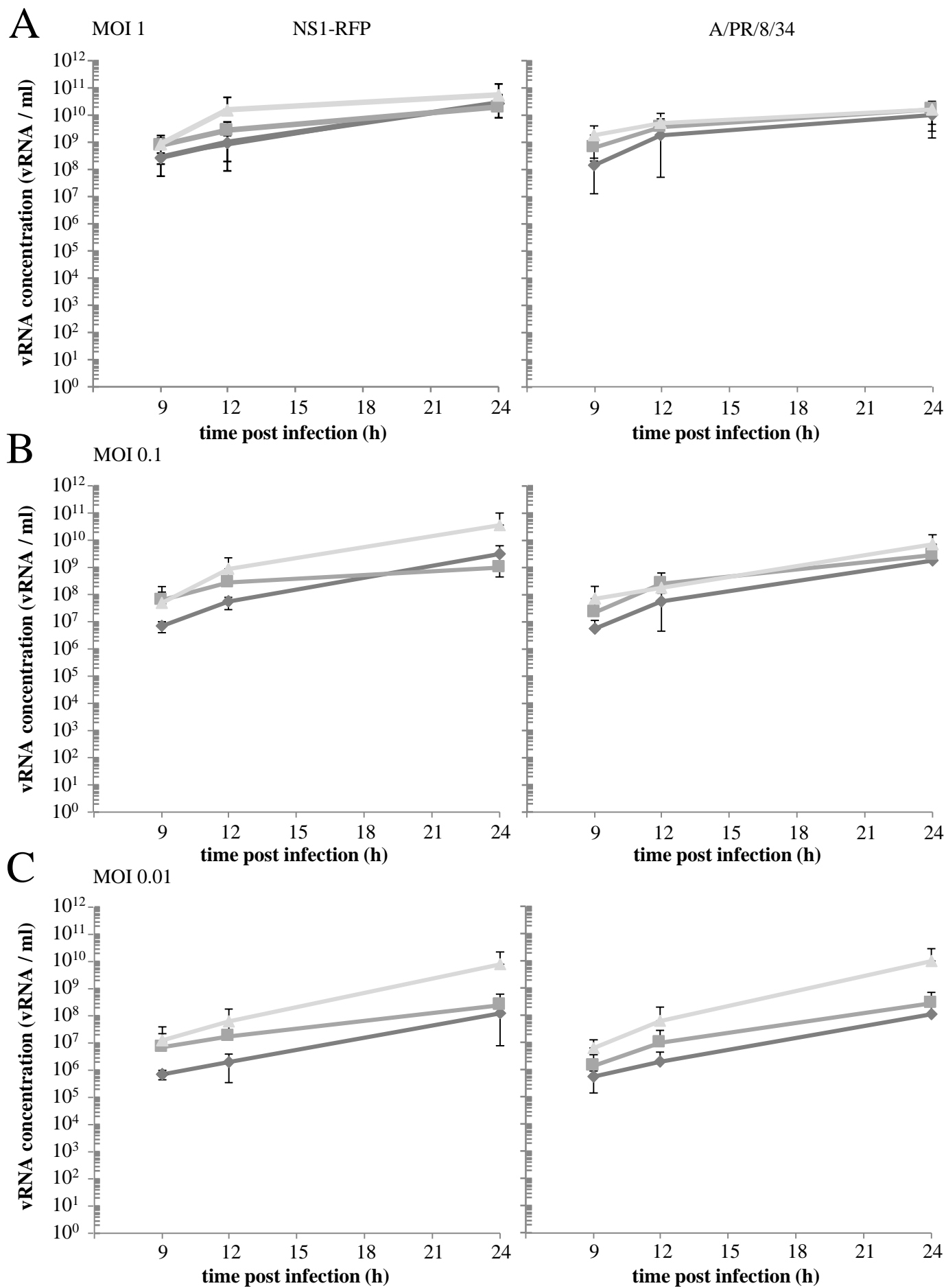

Figure S4

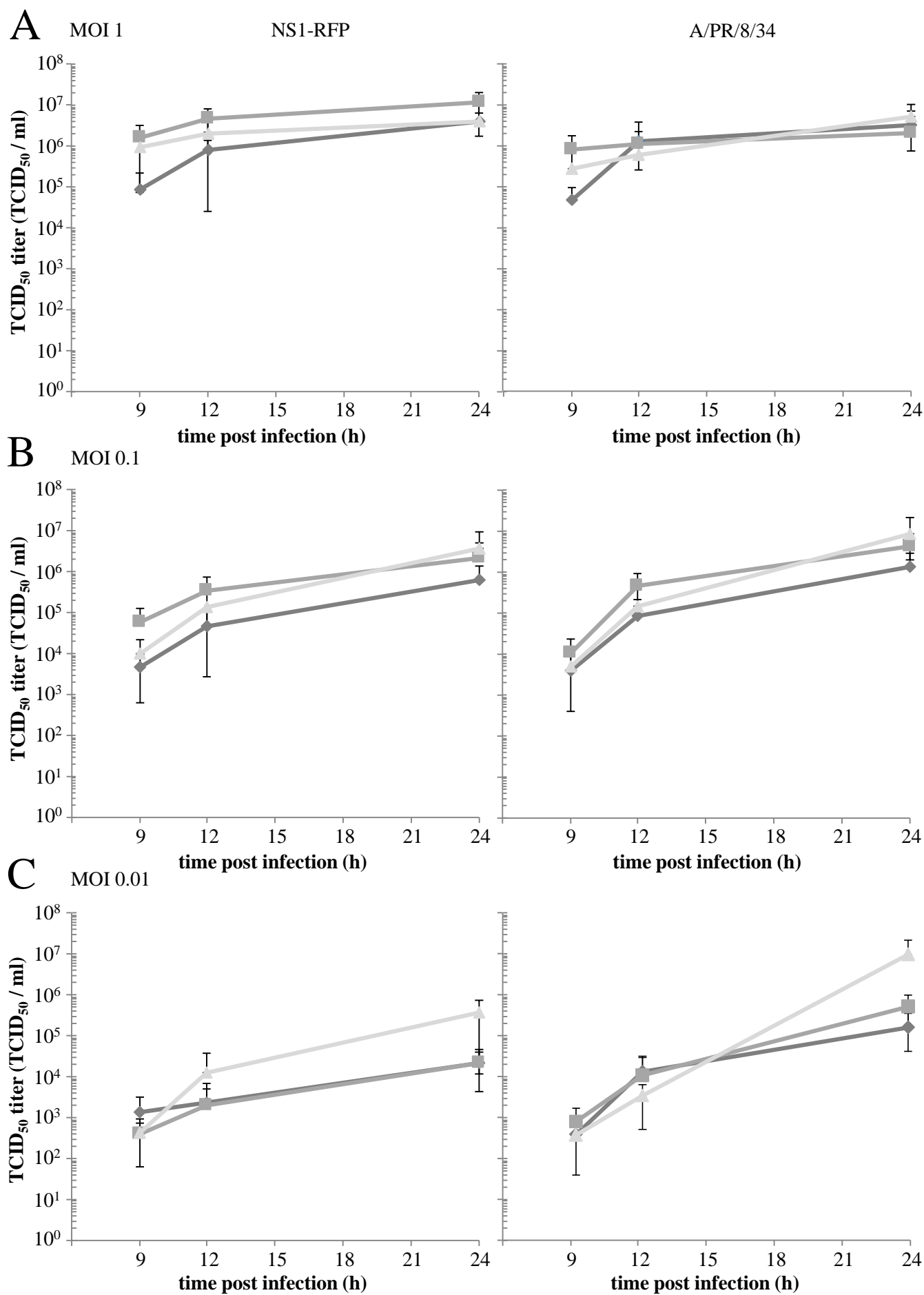

Figure S5

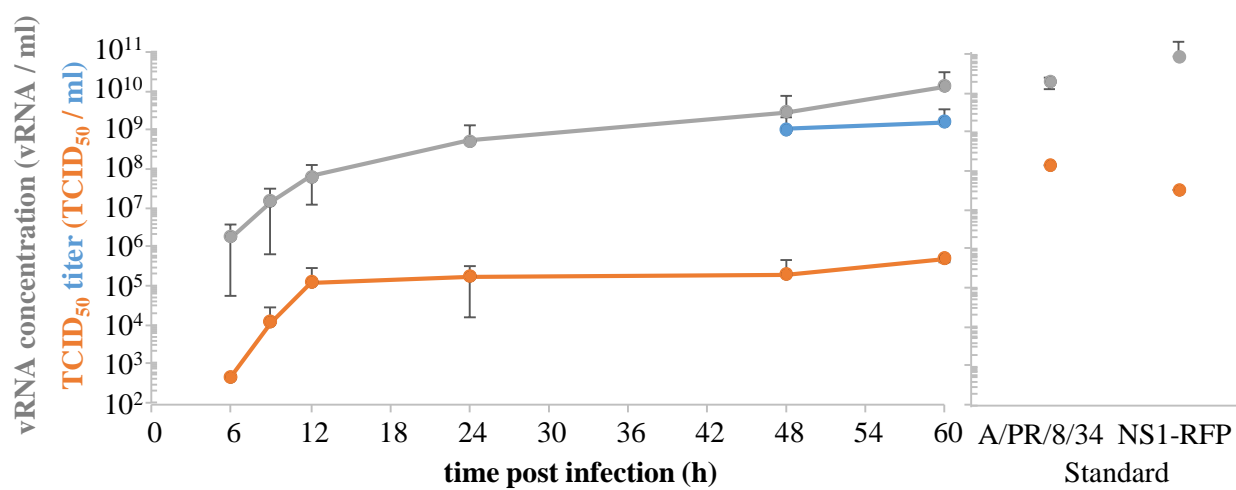

Figure S6

## 3D submerged model

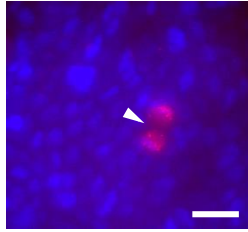

Figure S7

Supplement: Supplementary file 1 [file cells-11-03634-s001.zip › Suppl. Figures.pdf]
